# Reproductive differences among species, and between individuals and cohorts, in the leech genus *Helobdella* (Lophotrochozoa; Annelida; Clitellata; Hirudinida; Glossiphoniidae), with implications for reproductive resource allocation in hermaphrodites

**DOI:** 10.1371/journal.pone.0214581

**Published:** 2019-04-01

**Authors:** Roshni G. Iyer, D. Valle Rogers, Michelle Levine, Christopher J. Winchell, David A. Weisblat

**Affiliations:** 1 Dept. of Electrical Engineering & Computer Sciences, Univ. of California, Berkeley, CA, United States of America; 2 Dept. of Molecular & Cell Biology, Univ. of California, Berkeley, CA, United States of America; Universitaire Ziekenhuizen Leuven, BELGIUM

## Abstract

Leeches and oligochaetes comprise a monophyletic group of annelids, the Clitellata, whose reproduction is characterized by simultaneous hermaphroditism. While most clitellate species reproduce by cross-fertilization, self-fertilization has been described within the speciose genus *Helobdella*. Here we document the reproductive life histories and reproductive capacities for three other *Helobdella* species. Under laboratory conditions, both *H*. *robusta* and *H*. *octatestisaca* exhibit uniparental reproduction, apparently reflecting self-fertility, and suggesting that this trait is ancestral for the genus. However, the third species, *H*. *austinensis*, seems incapable of reproduction by self-fertilization, so we inferred its reproductive life history by analyzing reproduction in breeding cohorts. Comparing the reproductive parameters for *H*. *robusta* reproducing in isolation and in cohorts revealed that reproduction in cohorts is dramatically delayed with respect to that of isolated individuals, and that cohorts of leeches coordinate their cocoon deposition in a manner that is not predicted from the reproductive parameters of individuals reproducing in isolation. Finally, our comparisons of reproductive capacity for individuals versus cohorts for *H*. *robusta*, and between different sizes of cohorts for *H*. *austinensis*, reveal differences in resource allocation between male and female reproductive roles that are consistent with evolutionary theory.

## Introduction

Leeches comprise a monophyletic group of segmented worms within the phylum Annelida. They occupy primarily freshwater habitats, as fluid-feeding ectoparasites on vertebrate hosts, or predators or scavengers of freshwater invertebrates [1]. Molecular evidence indicates that leeches evolved from within the oligochaete annelids; together, these two taxa comprise the monophyletic assemblage of clitellate annelids [2–7]. Compared to oligochaetes, leeches are characterized by having lost segmentally iterated bristles (chaetae), by having a fixed number of segments, and by the presence of anterior and posterior suckers used for feeding and locomotion.

Certain leech species, primarily of the genus *Hirudo*, have proved valuable for analyzing neural circuits and behavior in terms of the activity and connectivity of individually identified neurons and for studies of individually defined neural cell types in culture [8]. Other species, primarily in the family Glossiphoniidae, have been used in studies of cell lineage and embryonic development, speciation, predator-prey interactions and genome evolution in the superphylum Lophotrochozoa [1, 9–12]. Thus, leeches generally, and those species in the glossiphoniid genus *Helobdella* in particular, provide models for integrating the questions and approaches from a wide range of biological sub-disciplines, from physiology and development to ecology, genomics and evolution in a less well explored branch of animals.

Leeches of the genus *Helobdella* are medium-sized (typically 1–3 cm as adults), neutrally pigmented, unobtrusive clitellate annelids, preying or scavenging on other invertebrates in shallow freshwater habitats. *Helobdella* and other glossiphoniid leeches are characterized by a large parental investment in reproduction [1, 13]. First, they produce relatively small numbers of large, yolk-rich eggs, ranging in diameter from 400 microns in *Helobdella* to roughly 2000 microns in *Haementeria ghilianii*. Second they exhibit a remarkable and complex brooding behavior. Internally fertilized eggs are deposited as meiotically arrested zygotes into cocoons on the parental venter, which folds to provide a protective concavity for the cocoons; then after the developing embryos hatch from their fertilization envelopes and the cocoons, they remain attached to the parental venter and are carried by the parent to one or more of their first meals, a cumulative period of time that can last many weeks.

Molecular-phylogenetic analyses have revealed a surprising diversity of the genus *Helobdella*: more than 50 species to date, many of which are difficult to distinguish morphologically [14–17]. In the course of ongoing studies using different *Helobdella* species for studying embryonic development in Lophotrochozoa/Spiralia, we have observed differences in reproduction, feeding, and other behaviors. We have previously described the reproductive life history of a self-fertile *Helobdella* species identified as *H*. *triserialis* [18]. The data presented here detail our findings concerning the reproductive life history, under similar conditions of laboratory culture, for *H*. *robusta* [19] and for a scute-bearing (*H*. *stagnalis*-like) species that we identify as *H*. *octatestisaca* [20] on the basis of its cytochrome oxidase 1 (CO1) sequence. Like *H*. *triserialis*, both of these species are self-fertile, as has also been reported for other glossiphoniid and piscicolid species [21–23]. Thus, we were surprised to discover that a third species, *H*. *austinensis* [24], is incapable of reproduction by self-fertilization. For this species we therefore inferred the reproductive life history of individuals by analyzing reproduction in breeding cohorts. This led us to compare the reproductive parameters for *H*. *robusta* raised in isolation and in cohorts, which yielded another surprising result. We found that reproduction by cohorts of *H*. *robusta* is dramatically delayed with respect to that of isolated individuals, and that cohorts of leeches coordinate their cocoon deposition in a manner that is not predicted from the reproductive parameters of isolated individuals.

This work is significant in several ways. First, reproduction by self-fertilization is uncommon even within hermaphroditic groups such as clitellate annelids, and may contribute to the extensively rearranged genome, speciosity and geographical distribution of the genus *Helobdella* [12, 25–27]. A capacity for self-fertilization means that rare transfer of even single individuals (for example by aquatic birds) could lead to the population of isolated habitats, and thus to new speciation events.

In addition, our observations show a correlation between reproductive activity and population density that suggests the utility of *Helobdella* as a system for testing theoretical predictions about resource allocation between egg and sperm production. In particular, our observations are consistent with the hypothesis that decreased opportunities to fertilize other individuals’ eggs with energetically lower cost sperm correlates with an increased production of eggs for animals at lower population densities. This correlation extends to the extreme case of isolated individuals for self-fertile species.

## Materials and methods

### Animals

**Fig 1:**
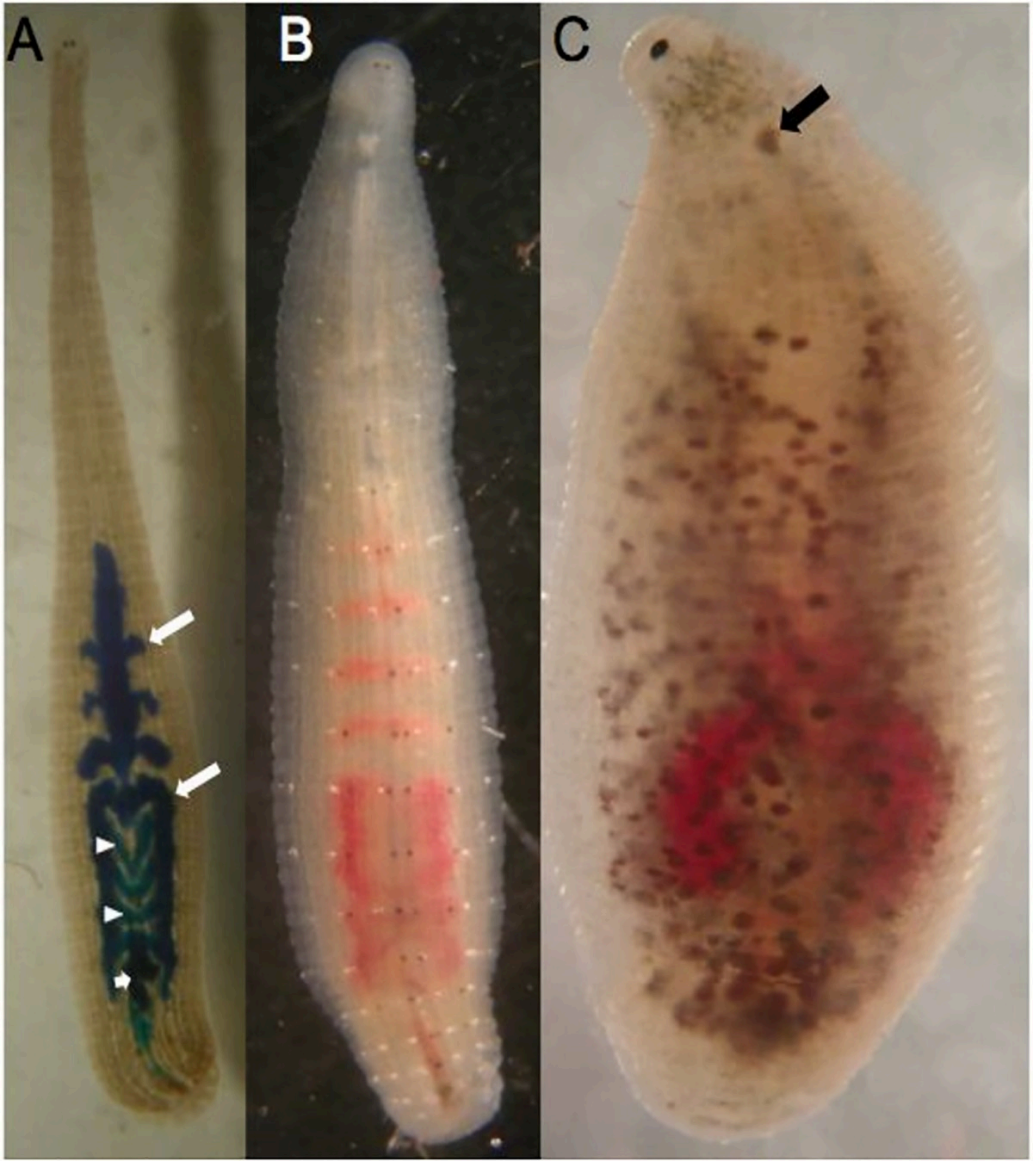
Three species of *Helobdella*. Dorsal views, anterior up, of adult *H*. *robusta*, *H*. *austinensis* and *H*.*octatestisaca*, respectively, highlighting differences in body wall pigmentation. A) This specimen had fed recently on an artificial food source containing Fast Green dye, which clearly outlines four of the five pairs of large anterior midgut lobes (caecae, long arrows), along with the four pairs of smaller intestinal lobes (arrowheads), and the rectum (short arrow). B) In this animal, which had fed on bloodworms, the crop caecae are labeled red; the central annulus in each segment contains prominent white and brown pigment patches. C) In common with other *H*. *stagnalis*-like species, this animal bears a chitinous scute (arrow) on the dorsal anterior surface.

The taxonomy of the genus *Helobdella* is in flux, due in large part to the increased resolution provided by the advent of molecular sequence comparisons. Data presented here represent three operational taxonomic units (OTUs; Fig 1):

OTU1 is the recently described *H*. *austinensis* (Hau) [24], collected from the wild in Austin, TX, and in continuous laboratory culture since 1997. OTU2 is *H*. *robusta* (Hro) [19], re-collected from its type location in Sacramento, CA and cultured in the laboratory for duration of this study (from approximately July, 2013 through July 2014). OTU3, collected from the same location as OTU2, and maintained in continuous laboratory culture is a *H*. *stagnalis*-like species, as defined by the presence of a nuchal scute on the dorsal surface at the boundary between the rostral and midbody segments. Molecular phylogenies have revealed that the morphologically defined *H*. *stagnalis* is in fact a complex of species [15, 28, 29]; CO1 sequencing indicates that the species used here is *H*. *octatestisaca* (Hoc) [20].

For comparison, we have also summarized previously published data [18], on the reproductive life history from a fourth OTU, *H*. *triserialis* (Htr; S2 Table). This taxon was originally collected in San Francisco, CA in the 1970s, and was recollected from the same site on various occasions; it was maintained in laboratory culture for several years through the early 1980s, but was lost from the laboratory and disappeared from its original location. CO1 sequence was obtained in 2006 from frozen specimens [14] (GenBank accession number DQ995303).

### Cytochrome c oxidase 1 (CO1) sequencing

For OTU1-3, a fragment of the mitochondrial CO1 gene was amplified and sequenced using standard procedures [14], using forward primer LCO1490 (5’-ggtcaacaaatcataaagatattgg-3’) and reverse primer HCO2198 (5’-taaacttcagggtgaccaaaaaatca-3’) [30]. Template DNA was extracted (Gentra Genomic DNA Purification Kit) from a single clutch of embryos that were removed from a leech of the appropriate species in the laboratory colony. These embryos were cultured in vitro by standard procedures to the stage at which they had exhausted all the yolk from their guts, but had not consumed any prey (unfed juvenile stage; [31, 11]. CO1 sequences for the species used here are available as GenBank accession numbers: Hau, MH729328; Hro, MH729330; Hoc, MH729329).

### Phylogenetic analysis

To estimate the phylogenetic positions of the three new OTUs studied here, and to examine the distribution of self-fertilization and scute presence within *Helobdella*, we first selected various *Helobdella* COI sequences from GenBank. For outgroups, we chose COI sequences from several other glossiphoniid genera (*Haementeria*, *Hemiclepsis*, *Theromyzon*) as well as a more distant piscicolid relative (*Zeylanicobdella*). We aligned the sequences with Clustal Omega [32] in Jalview [33] and used jModelTest [34] to select the model of sequence evolution (GTR+I+Γ) that best fit our data set. Using this model and empirical nucleotide frequencies, we constructed a Maximum Likelihood tree with PhyML 3.1 [35] using a BioNJ starting tree, 10 random starts, and NNI+SPR topology searching. Nodal support was calculated in PhyML with SH-like approximate likelihood ratio tests [36], and we used FigTree and Inkscape software to edit the tree for presentation.

### Reproductive analysis

For some experiments, individuals for which the exact birthdate (defined here as the date of zygote deposition into cocoons on the parental venter) was known were reared in isolation from early stages of development in small petri dishes (35 or 50 mm diameter), with daily feeding and changes of water (1/100 dilution of artificial seawater; Salinity for Reefs, Aquavitro) at room temperature (21–23°C). For other experiments, groups of late stage embryos or early juveniles, from a clutch for which the exact birth date was known, were isolated and reared as freely breeding cohorts, maintained as above except for being transferred as adults to larger containers (0.5–1 liter capacity pyrex bowls). With rare exceptions, animals in both conditions were checked daily for reproductive activity and deaths. Cocoons were removed and embryos enumerated as described elsewhere [31]; embryos were usually removed and counted within 24 hours of zygote deposition. In apparent contrast to the situation with *Hirudo* [1], essentially all the *Helobdella* zygotes developed normally except for those damaged during removal from the parent. On occasions where clutch deposition was not observed immediately, the date of laying was estimated from the stage of development attained when the clutch was removed.

### Computer simulations of reproductive activity

Cohort breeding behavior was modeled using a Monte Carlo simulator with an automated graph plotter (details of the program and instructions for use are available at: https://github.com/roshnigiyer/Monte_Carlo_Simulator). Separate sets of model parameters were derived from life history data of *H*. *robusta* reared in isolation and in cohorts, including mean and standard deviation measures for time-to-first-clutch, inter-clutch intervals, clutch size, number of clutches laid, days survived after last clutch and lifespan. Our Monte Carlo simulator generates these data in under 1 second on average using 100 simulation runs.

## Results and discussion

### Reproductive life histories of individuals raised in isolation differ among three self-fertile *Helobdella* species

Sexual reproduction by simultaneous hermaphrodites is the presumed ancestral state of clitellate annelids, although some species now rely in part or entirely on various modes of asexual reproduction [37, 38]; the ecology of sexual reproduction has been reviewed [39]. Cross-fertilization is required for most clitellate annelids, but several species have the ability to produce viable embryos without ever having had contact with prospective mates. While the possibility of other uniparental modes of reproduction has not been rigorously excluded, polar body formation, indicative of maternal meiosis, has been observed in uniparental zygotes [40]; DAW unpublished observations), and this capacity for reproducing without mating in leeches has generally been accepted as self-fertilization. Thus, we will use that term here. By this criterion, self-fertilization among leeches has been documented previously for the piscicolid species *Zeylanicobdella arugamensis* [21], and for at least three glossiphoniid species, including the species referred to by Whitman as *Clepsine marginata* [23]—now *Hemiclepsis marginata*, *Helobdella triserialis* [18] and *H*. *papillornata* [22]. Here, we document that the strains of *H*. *robusta* and *H*. *octatestisaca* that we have studied are self-fertile, as well as being capable of cross-fertilization. Individuals raised in isolation from embryonic stages routinely produce viable young, and these progeny are also self-fertile when reared in isolation. In these self-fertilizing animals, we found no evidence of the externally implanted spermatophores that are seen upon cross-fertilization in these species. Thus, we conclude that self-fertilization does not involve implantation of a spermatophore, but rather is achieved internally.

For these self-fertile species, as for *H*. *triserialis* [18], it was possible to directly measure the reproductive capacity (defined here as the number of young produced during the life of one individual) under defined conditions by rearing individuals in isolation from early stages of development until their death, removing and determining the size of all clutches for each individual. For comparison, previously published comparable data for *H*. *triserialis* [18] is summarized here as well.

Previous work had shown that, when reared in isolation under laboratory conditions at room temperature, *H*. *triserialis* exhibits an egg-to-egg generation of time of about 70 days, then generates five clutches of embryos at 30–35 day intervals. Of these five clutches, the first and last were smaller and the third was the largest (S2 Table). For *H*. *triserialis* reared in isolation under these conditions, five clutches was a hard maximum; at least one individual survived for over three months after depositing its fifth clutch, well beyond the average inter-clutch interval, without further reproduction, and the production of fewer than five clutches was invariably associated with premature death of the animal. The average reproductive capacity measured for *H*. *triserialis* in those experiments was 302 offspring per individual.

The reproductive life history for *H*. *robusta* is summarized in Tables 1 and 2 (for more detailed information see S3 Table) differs both qualitatively and quantitatively from that previously described for *H*. *triserialis* under similar conditions. Firstly, the average egg-to-egg generation time for this species was 57 days and the average inter-clutch interval was less than 30 days. In addition, this species was capable of laying more than five clutches of embryos; a maximum of eight was observed. This increase in the number of clutches was associated with somewhat smaller clutch sizes, and with a more uniform distribution of clutch sizes (compare Table 2, S2 and S3 Tables). The average reproductive capacity for *H*. *robusta* raised in isolation was 267 offspring per individual, with a maximum of 392.

**Table 1:** Clutch size data for self-fertilizing and interbreeding cohorts of *Helobdella* spp.

*H*. *robusta*: self-fertilizing, snail diet (N = 16)*		
Clutch number (S)**	**Clutch size**	**Clutch size (min, max)**
**C1 (10)**	20.3 +/- 4.1	(15, 25)
**C2 (14)**	51.1 +/- 16.3	(3, 70)
**C3 (14)**	71.1 +/- 19.5	(34, 99)
**C4 (11)**	77.6 +/- 19.9	(55, 104)
**C5 (11)**	69.1 +/- 23.5	(30, 105)
**C6 (9)**	51.0 +/- 30.7	(7, 95)
**C7 (6)**	44.2 +/- 27.3	(12, 81)
**C8 (1)**	13	(13, 13)
*H*. *octitestisaca*: self-fertilizing, bloodworm diet (N = 5)*		
Clutch number (S)**	**Clutch size**	**Clutch size (min, max)**
**C1 (5)**	26.4 +/- 7.5	(17, 37)
**C2 (5)**	50.2 +/- 12.7	(33, 66)
**C3 (3)**	70.3 +/- 4.7	(65, 74)
*H*. *austinensis*: interbreeding cohort, bloodworm diet (N = 23)*		
Clutch number (S)**	**Clutch size**	**Clutch size (min, max)**
**C1 (23)**	94.2 +/- 41.0	(45, 179)
**C2 (16)**	87.4 +/- 36.2	(22, 160)
**C3 (1)**	26	(26, 26)
*H*. *austinensis*: interbreeding cohort, bloodworm diet (N = 60)*		
Clutch number (S)**	**Clutch size**	**Clutch size (min, max)**
**C1 (60)**	43.8 +/- 20.8	(6, 117)
**C2 (54)**	41.5 +/- 22.4	(7, 93)
**C3 (31)**	38.9 +/- 25.2	(6, 97)
*H*. *robusta*: interbreeding cohort, snail diet (N = 48)*		
Clutch number (S)**	**Clutch size**	**Clutch size (min, max)**
**C1 (48)**	24.0 +/- 10.0	(11, 65)
**C2 (34)**	44.6 +/- 16.8	(15, 85)
**C3 (28)**	68.4 +/- 18.4	(21, 102)
**C4 (26)**	75.3 +/- 18.5	(29, 114)
**C5 (13)**	48.1 +/- 22.3	(17, 85)

Clutch size is average +/- standard deviation

*N is the number of individual animals, or the size of the cohort

**Sample size (S), is the number of actual or inferred clutches; for interbreeding cohorts, clutch number was inferred as described in text.

**Table 2:** Clutch interval data for self-fertilizing and interbreeding cohorts of *Helobdella* spp.

*H*. *robusta*: self-fertilizing, snail diet (N = 16)*			
Interval (S)**	**Zygote-clutch interval**	**Inter-clutch interval**	**Inter-clutch interval (min, max)**
**ZD-C1 (15)**	56.3 +/- 8.7		(37, 68)
**C1-C2 (15)**	83.5 +/- 7.7	28	(72, 93)
**C2-C3 (14)**	107.4 +/- 10.5	23	(92, 121)
**C3-C4 (12)**	136.8 +/- 9.9	30	(117, 150)
**C4-C5 (10)**	170.4 +/- 16.0	33	(142, 193)
**C5-C6 (8)**	199.4 +/- 17.2	29	(169, 222)
**C6-C7 (5)**	243.3 +/- 29.6	44	(217, 295)
**C7-C8 (1)**	264	21	(264, 264)
*H*. *octitestisaca*: self-fertilizing, bloodworm diet (N = 5)*			
Interval (S)**	**Zygote-clutch interval**	**Inter-clutch interval**	**Inter-clutch interval (min, max)**
**ZD-C1 (5)**	140.0 +/- 25.8		(120, 180)
**C1-C2 (4)**	160.8 +/- 37.7	21	(137, 227)
**C2-C3 (3)**	220.8 +/- 43.2	60	(191, 270)
*H*. *austinensis*: interbreeding cohort, bloodworm diet (N = 23)*			
Interval (S)**	**Zygote-clutch interval**	**Inter-clutch interval**	**Inter-clutch interval (min, max)**
**ZD-C1 (23)**	109.3 +/- 20.4		(80, 148)
**C1-C2 (16)**	188.4 +/- 36.6	79	(148, 240)
**C2-C3 (1)**	243	55	(243, 243)
*H*. *austinensis*: interbreeding cohort, bloodworm diet (N = 60)*			
Interval (S)**	**Zygote-clutch interval**	**Inter-clutch interval**	**Inter-clutch interval (min, max)**
**ZD-C1 (60)**	109.7 +/- 19.3		(70, 143)
**C1-C2 (54)**	189.8 +/- 28.3	80	(144, 234)
**C2-C3 (31)**	273.1 +/- 57.2	83	(237, 346)
*H*. *robusta*: interbreeding cohort, snail diet (N = 48)*			
Interval (S)**	**Zygote-clutch interval**	**Inter-clutch interval**	**Inter-clutch interval (min, max)**
**ZD-C1 (48)**	107.4 +/- 10.7		(94, 134)
**C1-C2 (34)**	146.8 +/- 11.1	40	(135, 170)
**C2-C3 (28)**	184.9 +/- 11.0	48	(171, 204)
**C3-C4 (26)**	221.1 +/- 13.2	36	(204, 241)
**C4-C5 (13)**	252.8 +/- 9.2	32	(241, 276)

Zygote-to-first-clutch and inter-clutch Intervals given as average +/- standard deviation.

*N is the number of individual animals, or the size of the cohort

**Sample size (S), is the number of actual or inferred clutches; for interbreeding cohorts, clutch number was inferred as described in text.

The reproductive life history we observed for isolated, self-fertilizing *H*. *octatestisaca* (summarized in Tables 1 and 2; more detailed information in S4 Table) differed markedly from those described for either *H*. *robusta* or *H*. *triserialis*. From among a sample of five individuals, the egg-to-egg generation time was longer (140 days versus 56 days for *H*. *robusta*), no individual produced more than three clutches of embryos, and the average reproductive capacity was dramatically less (119 versus 267 for *H*. *robusta*). Although we cannot exclude the possibility that these differences reflect culture conditions that were suboptimal for *H*. *octatestisaca*, the average lifespan of *H*. *octatestisaca* in this experiment (246 days) was not less than that of *H*. *robusta* under similar conditions (229 days), and several of the animals survived after laying their last clutch of embryos for periods of time that were much longer than the average inter-clutch interval. Moreover, the fact that both species were collected from the same site makes it seem less likely, though certainly not impossible, that they would respond so differently to a fixed set of laboratory culture conditions.

### *Helobdella austinensis* does not reproduce in isolation

Given that *H*. *triserialis*, *H*. *papillornata*, *H*. *robusta* and *H*. *octatestisaca* are all self-fertile, it came as a surprise that we were unable to observe self-fertilization for *H*. *austinensis*, which is more closely related to *H*. *robusta* than are the other three self-fertile species of *Helobdella* (S1 Fig). Individuals reared in isolation for months failed to reproduce, but soon became gravid when placed with other individuals (data not shown). Thus, to study the reproductive life history of *H*. *austinensis*, we raised cohorts of interbreeding animals, tracking survivorship and reproductive activity of the adult cohort by noting the dates of deaths and clutch depositions, and the size of clutches produced during the collective life of the cohort, respectively.

### Life history analyses for cohorts of interbreeding *H*. *austinensis* suggest differences in reproductive behaviors from other *Helobdella* species

**Fig 2:**
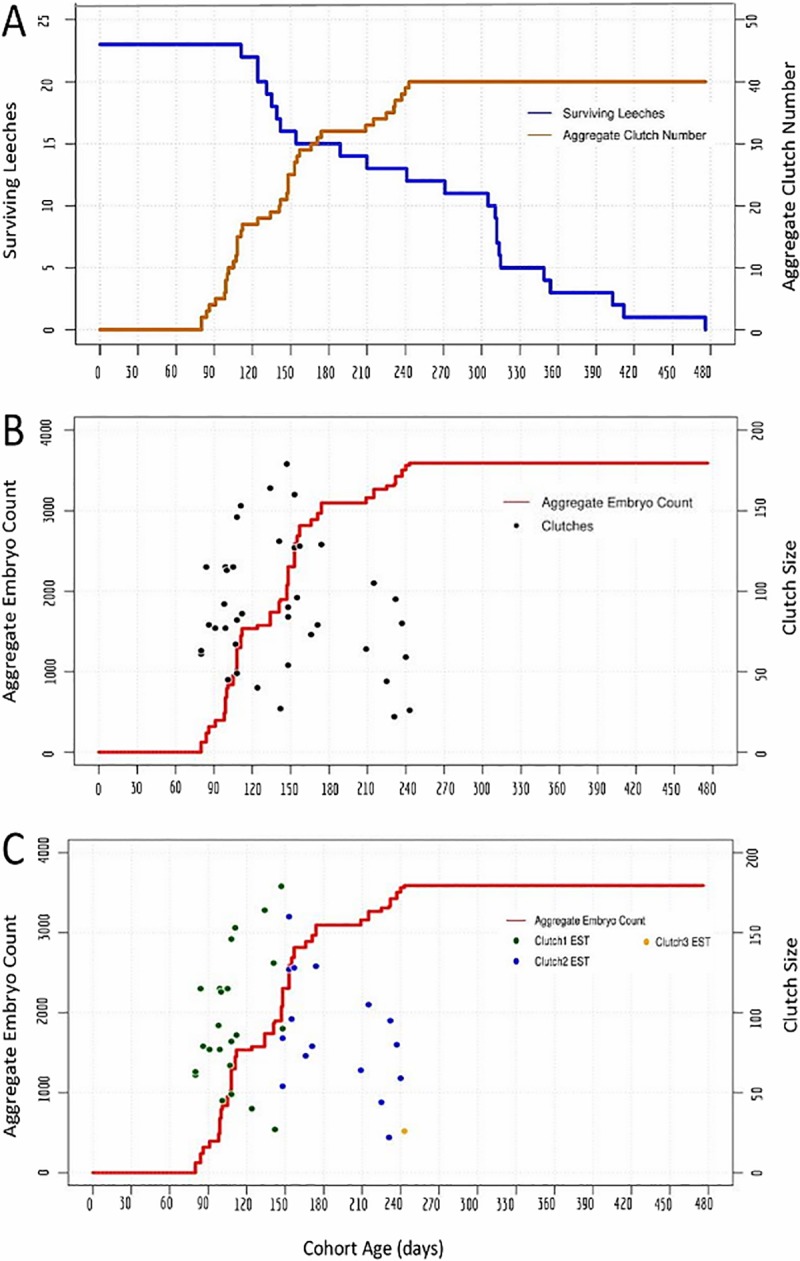
Reproduction in an interbreeding cohort of 23 *H*. *austinensis*. A) Cohort survival (blue, left axis) and aggregate clutch production (orange, right axis) as a function of time, for a cohort of animals fed ad lib on bloodworms. B) Aggregate embryo production (red, left axis); black dots indicate the size (number of embryos, right axis) and deposition date of each individual clutch. C) The same data as in B, except the estimated (EST) assignments of clutches into first, second and third layings are indicated by coloring dots as indicated (see text for details). 95% confidence intervals for the timing and clutch size of the inferred clusters of reproductive activity are: 100 to 118 days and 76 to 112 embryos for cluster 1; 169 to 208 days and 68 to 107 embryos for cluster 2.

**Fig 3:**
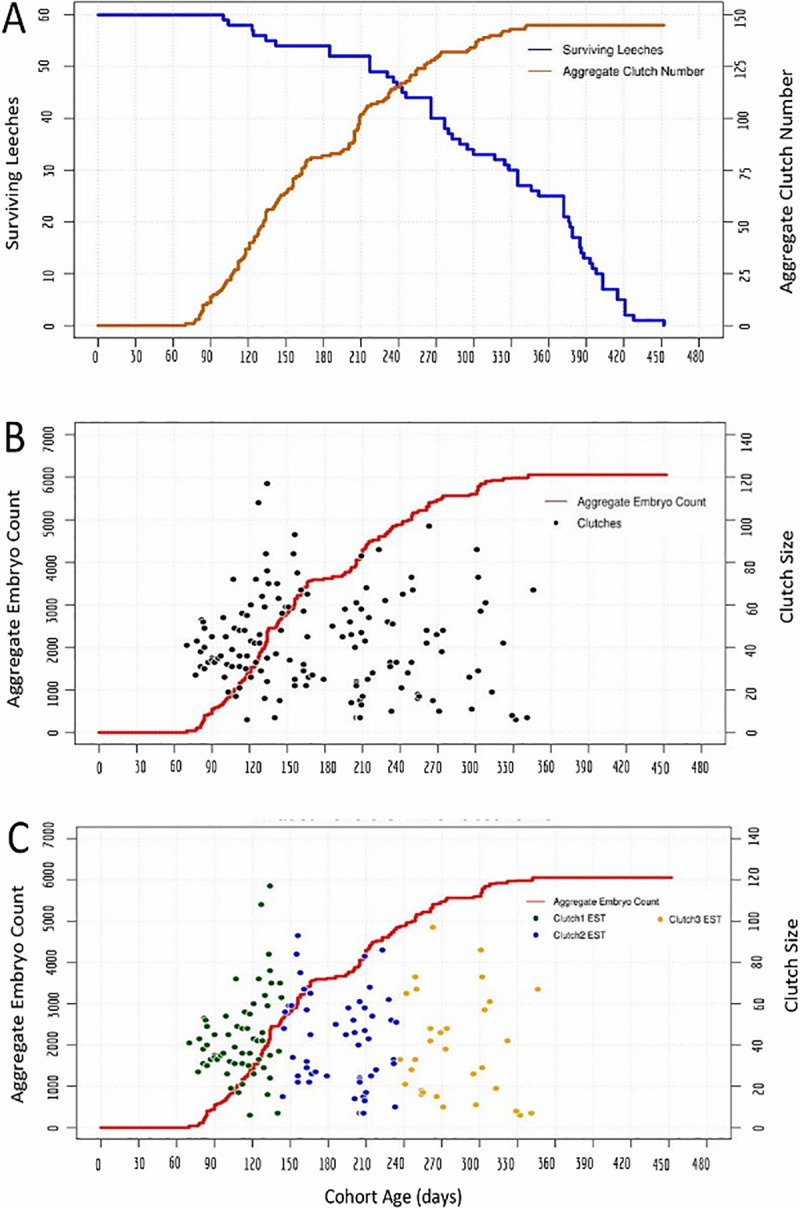
Reproduction in an interbreeding cohort of 60 *H*. *austinensis*. A) Cohort survival (blue, left axis) and aggregate clutch production (orange, right axis) as a function of time, for a cohort of animals fed ad lib on bloodworms. B) Aggregate embryo production (red, left axis); black dots indicate the size (number of embryos, right axis) and deposition date of each individual clutch. C) The same data as in B, except the estimated (EST) assignments of clutches into first, second and third layings are indicated by coloring dots as indicated (see text for details). 95% confidence intervals for the timing and clutch size of the inferred clusters of reproductive activity are: 107 to 115 days and 39 to 48 embryos for cluster 1; 211 to 217 days and 35 to 47 embryos for cluster 2; 269 to 293 days and 30 to 48 embryos for cluster 3.

We studied two cohorts of *H*. *austinensis* reared on a diet of commercially available "bloodworms" (frozen midge larvae); clutches of embryos were removed and counted as soon as they were observed, usually within 24 hours of having been deposited. The two cohorts, starting with 23 and 60 individuals, respectively, produced a total of 40 and 145 clutches, respectively. For each experiment, we tracked the number of surviving leeches within the cohort, the number of clutches deposited, the number of embryos per clutch and the aggregate number of embryos produced (Figs 2 and 3).

The reproductive behavior of individuals within the two cohorts was inferred based on two assumptions: 1) that leeches raised under similar conditions breed in rough synchrony, and 2) that all individuals in the cohort reproduce. Based on these assumptions, we defined the first round of egg laying as beginning with deposition of the first clutch of embryos and ending when the number of clutches deposited equaled the number of animals that had been present when the first clutch was deposited. Similarly, the second and subsequent rounds of reproduction were defined as beginning with the deposition of the next new clutch and ending when the number of additional clutches produced equaled the number of animals that had been present at the beginning of that round of reproduction.

This analysis is subject to various possible errors. For example, if any animals die during the first reproductive round without having produced a clutch of embryos, then what we define as that round would be extended artifactually to include clutches that are actually part of the second round. Conversely, if the temporal spread of reproductive activity is large, the last clutches deposited in the first round of egg laying might be assigned to the second round and vice versa, which would artifactually shorten what we define as the first round. Finally, notwithstanding the fact that all the animals are simultaneous hermaphrodites, we cannot exclude the possibility that some individuals in this experiment were not inseminated and thus failed to deposit zygotes in a given round of reproduction.

Applying this analysis to data obtained for the first cohort experiment (starting with 23 individuals) suggests that most animals in the cohort underwent two rounds of reproduction (Fig 2), consisting of 23 and 16 clutches and centered at 109 and 188 days after the birth of the cohort, respectively (Table 2). Based on the assumptions described above, only a single clutch of embryos was assigned to a putative third round of reproduction (Fig 2C; Tables 1 and 2). On the other hand, the gap in reproductive activity of the cohort between 175 and 208 days, followed by a cluster of layings between 209 and 240 days, could mean that one of our initial assumptions was in error, and that eight animals underwent a third round of reproduction, from among the 13 surviving at 210 days. In either case, no layings occurred after 243 days, despite the fact that the last individuals in the cohort survived for well over 100 days after the last clutch was deposited. Thus, we concluded that three rounds of reproduction were the maximum observed in this experiment, if the starting assumptions hold true.

The second cohort experiment started with a cohort of 60 *H*. *austinensis* reared under similar conditions to the first (Fig 3A and 3B). Interpreting the data from this cohort under the assumptions introduced above again indicates a maximum of three rounds of reproduction, consisting of 60, 54, and 31 clutches and centered at 110, 190 and 273 days after the birth of the cohort, respectively (Fig 3C; Table 2). No further embryos were produced during the last 105 days of the experiment (day 347 through 452), despite the fact that there were 25 surviving individuals at the start of this period. Thus, we again concluded that no individual of *H*. *austinensis* produced more than three clutches of embryos under these conditions.

### Possible environmental influences on reproductive behavior in *H*. *austinensis*

Clutch sizes in the two *H*. *austinensis* cohort experiments varied widely, from 6 to 179 embryos. There was no significant difference between the average size of the inferred first and second clutches *within* either experiment (Table 1). Surprisingly, however, the first two clutches in the first cohort experiment averaged more than twice the size of the corresponding clutches in the second cohort experiment (Table 1). Moreover, the average reproductive capacity in the first experiment (3591 embryos/23 individuals; 156 embryos/individual) was also larger than that in the second experiment (6055 embryos/60 individuals; 101 embryos/individual), despite the fact that more individuals in the second cohort appeared to have laid third clutches of embryos. Animals in both cohort experiments were fed *ad libitum* and no obvious size differences between specimens in the two cohorts were noted. Thus, it seems unlikely that competition for food among the larger cohort is responsible for the difference.

In any event, two considerations lead us to conclude that these values are conservative estimates of reproductive capacity. First is the likelihood that some animals in the cohort die without exhausting their reproductive capacity. Premature death could result from disease induced by sub-optimal culture conditions or inadvertent damage while removing embryos for counting. Another factor is the likely influence of diet on growth and reproduction. *Helobdella* species maintained in our lab are fed frozen chironomid insect larvae (bloodworms), and/or live snails (primarily *Lymnaea* and *Physa*); the three species of *Helobdella* studied here exhibit different dietary preferences. *H*. *octatestisaca* strongly prefer bloodworms; snails placed in their bowl survive for many days as long as bloodworms are provided. In contrast, *H*. *robusta* exhibit a strong preference for snails; we have not succeeded in maintaining this species on a pure bloodworm diet. Finally, *H*. *austinensis* feed and breed readily on either bloodworms or snails, but grow much larger when fed snails. Individuals fed with excess bloodworms seldom exceed 40 mg in size (R. Kim, personal communication), and the maximum clutch size for animals on a bloodworm diet was 179 embryos (Table 1); in contrast, snail-fed individuals can grow to more than 120 mg and produce single clutches of over 200 embryos (S. Yoo, personal communication). Unfortunately, a systematic investigation of the links between diet and reproductive capacity was beyond the scope of the present work. Technical limitations, including the inability to reliably procure adequate numbers of snails, prevented us from carrying out a systematic comparison of the reproductive parameters of *H*. *austinensis* reared on snails versus bloodworms.

### Breeding cohorts of *H*. *robusta* exhibit temporally clustered bouts of reproduction

The indirect conclusion that *H*. *austinensis* exhibits a maximum of three bouts of reproduction was similar to our observations based on direct observation of reproductive behaviors of self-fertilizing individual *H*. *octatestisaca* individuals, but markedly different than for what we observed for self-fertilizing individual *H*. *triserialis* (up to five layings) and *H*. *robusta* (up to eight egg layings) (S2 Table; Tables 1 and 2). The strength of these inter-species comparisons is limited, however, by the differences in the experimental conditions—some of the observed differences might reflect differences between animals in interbreeding cohorts versus self-fertilizing animals in isolation.

It is obviously not possible to observe the reproductive behavior of individual *H*. *austinensis* in isolation. Thus, to compare the reproductive behavior of a cohort of leeches to that of isolated conspecifics, we re-collected *H*. *robusta* from the type location [24], confirmed the species identity by CO1 sequencing (see Materials and Methods), and then carried out a cohort breeding study starting with animals originating as a single clutch of embryos. The experiment was carried out as described above for *H*. *austinensis* except that *H*. *robusta* were fed on their preferred diet of live, lab-reared snails, as for the experiments on isolated, self-fertilizing *H*. *robusta*.

**Fig 4:**
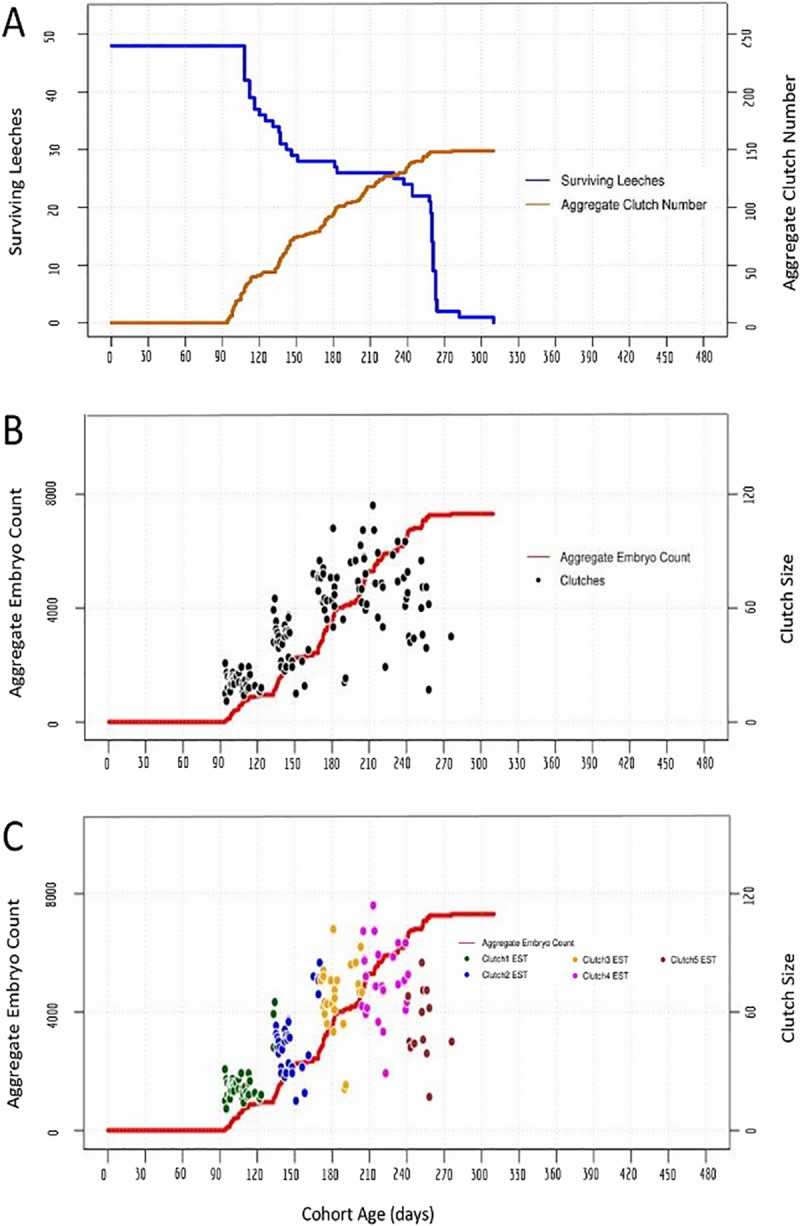
An interbreeding cohort of 48 *H*. *robusta* exhibits clustered bouts of reproduction. A) Cohort survival (blue, left axis) and aggregate clutch production (orange, right axis) as a function of time, for a cohort of animals fed ad lib on snails. B) Aggregate embryo production (red, left axis); black dots indicate the size (number of embryos, right axis) and deposition date of each individual clutch. C) The same data as in B, except the estimated (EST) assignments of clutches into first, second and third layings are indicated by coloring dots as indicated (see text for details). 95% confidence intervals for the timing and clutch size of the inferred clusters of reproductive activity are: 104 to 110 days and 21 to 27 embryos for cluster 1; 143 to 151 days and 40 to 51 embryos for cluster 2; 180 to 189 days and 61 to 73 embryos for cluster 3; 216 to 226 days and 68 to 83 embryos for cluster 4; 248 to 258 days and 43 to 63 embryos for cluster 5.

**Fig 5:**
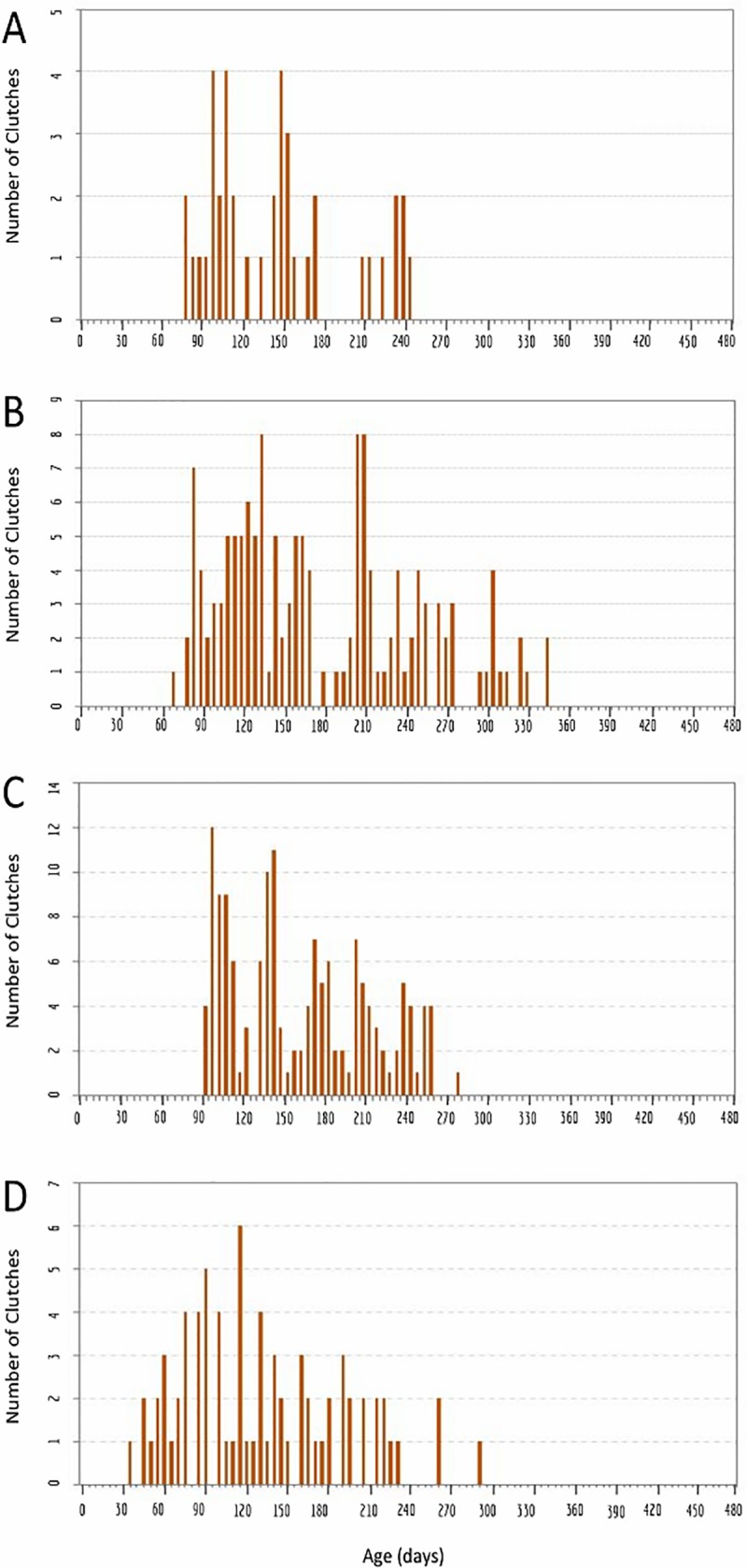
Bar graph showing the distribution of clutch deposition. Clutch deposition events occurring within five day bins, for the experiments shown in Figs 2, 3, 4 and 6.

Starting with a cohort of 48 animals, a total of 7304 embryos from 149 clutches were produced ranging in size from 11 to 114 embryos (Tables 1 and 2; Fig 4A and 4B). Casual inspection of the data suggested several differences between reproductive behavior in *H*. *austinensis* and *H*. *robusta*: 1) bouts of reproductive activity in the *H*. *robusta* cohort were more tightly clustered than for *H*. *austinensis* in both timing and clutch size (Fig 5); 2) there appeared to be five such bouts, and; 3) there was an apparent correlation between clutch size and reproductive episode, with the third and fourth clutches being the largest. There was no significant difference in the egg-to-egg generation time between the two species under these conditions (Table 2).

Analyzing the reproductive behavior data under the same assumptions as for *H*. *austinensis* suggested that the cohort reproduced in five clusters of 48, 34, 28, 26 and 13 layings, respectively (Figs 4C and 5; Tables 1 and 2). There was a sharp drop in population after the fifth cluster of reproductive activity, and the last surviving leech in the cohort died only 34 days after the last clutch of embryos was laid. This sharp decline contrasts with the gradual decline and extended post-reproductive survival of individuals in the two cohort experiments for *H*. *austinensis* (cf Figs 1 and 2).

One interpretation is that this difference in observed lifespan reflects interspecific differences in developmental time and/or post-embryonic parental care between *H*. *robusta* and *H*. *austinensis*. For example, *H*. *robusta* develops to the juvenile stage in about 10 days, whereas *H*. *austinensis* requires about 13 days (S. Yoo et al. in preparation). The length of time that individual young remain on the parent in both species is highly variable and has not been analyzed systematically. Another possibility is that the *H*. *robusta* cohort died off prematurely, either due to parasites picked up from the snails, or to other, unknown factors. Problems of colony decline and extinction have been noted by ourselves and others for *H*. *triserialis* and *H*. *robusta* (M. Shankland, personal communication; D.H. Kuo, personal communication), and are responsible for the shift to using *H*. *austinensis* as a more lab-tractable species for study. Our present data do not allow us to distinguish rigorously between these possibilities.

### Reproduction parameters derived from isolated *H*. *robusta* do not predict the clustered bouts of reproductive activity seen in breeding cohorts

**Fig 6:**
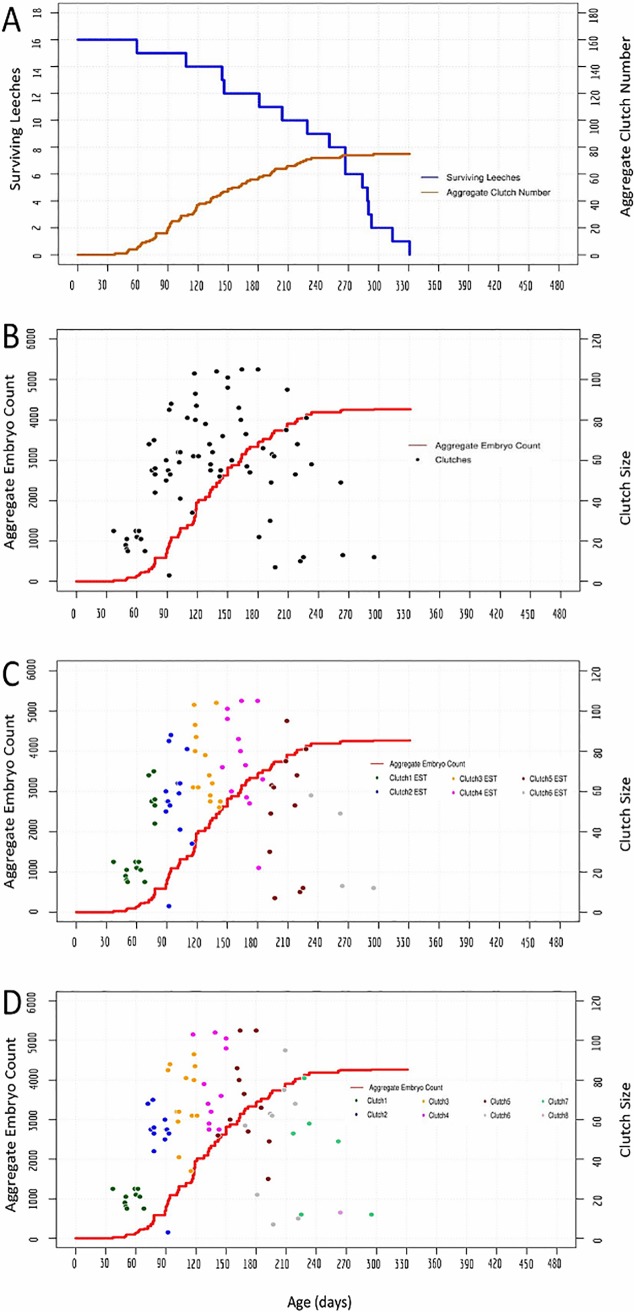
Clustered reproductive activity by a *H*. *robusta* cohort is not observed in a “pseudo-cohort”. The pseudo cohort was generated by combining the reproductive parameters observed for isolated individuals. A-C) A pseudo-cohort was created by graphing the aggregated data from 16 animals reared in isolation. 95% confidence intervals for the timing and clutch size of the inferred clusters of reproductive activity are: 56 to 70 days and 24 to 45 embryos for cluster 1; 93 to 101 days and 47 to 70 embryos for cluster 2; 122 to 134 days and 65 to 84 embryos for cluster 3; 158 to 177 days and 59 to 86 embryos for cluster 4; 199 to 219 days and 29 to 71 embryos for cluster 5; 223 to 304 days and 0 to 71 embryos for cluster 6. D) For comparison, the actual clutch groupings are denoted using the same color scheme. 95% confidence intervals for the timing and clutch size of the actual clusters of reproductive activity are: 49 to 61 days and 18 to 23 embryos for cluster 1; 79 to 88 days and 42 to 62 embryos for cluster 2; 102 to 113 days and 60 to 80 embryos for cluster 3; 131 to 143 days and 65 to 90 embryos for cluster 4; 160 to 181 days and 54 to 84 embryos for cluster 5; 187 to 212 days and 29 to 73 embryos for cluster 6; 216 to 271 days and 19 to 69 embryos for cluster 7. Note that the inference procedure misassigned some clutches, and that only six rounds of reproduction were inferred, whereas the true value was eight.

To compare the reproductive behaviors of *H*. *robusta* in isolation and in cohorts, we first plotted the combined reproductive data from 16 isolated individuals, comprising a total of 75 clutches, to ask how well the resultant “pseudo-cohort” data recapitulated the reproductive behavior of the true cohort of 48 individuals (Fig 6A and 6B). For this dataset, we could also determine how well the reproductive behaviors inferred using the assumptions applied to the actual cohorts of *H*. *austinensis* (Fig 6C) match the known reproductive behavior of the individuals comprising the pseudo-cohort (Fig 6D).

Surprisingly, the pseudo-cohort data (Fig 6) differ from the cohort data (Fig 4) in at least two ways. First, the onset of reproductive activity in isolated animals was markedly earlier than in the cohort experiment. Among animals raised in isolation, the earliest reproductive episode occurred just 37 days into the life of the parent and all 16 individuals had completed their first round of reproduction by 68 days (Table 2); indeed, most animals reared in isolation had completed their *second* round of reproduction before the first cluster of reproductive activity among the cohort animals. Second, the discrete clusters of reproductive activity in the cohort were largely absent from the pseudo-cohort, especially after the first bout of reproduction. A third difference is that the cohort seemed to undergo a maximum of five rounds of reproduction, whereas isolated individuals deposited up to eight clutches of embryos. As mentioned above, however, we cannot exclude the possibility that the cohort population died off before exhausting its reproductive capacity. Moreover, comparing the inferred clutch groupings (Fig 6C) with the actual clutch groupings (Fig 6D) shows that the inference method predicted a wider temporal distribution of clutch deposition times and a reduced number of egg layings (six) relative to the actual data (eight).

### Computer simulations of reproductive activity

As a further inquiry into the apparent difference in reproductive activity between isolated individuals and an interbreeding cohort of *H*. *robusta*, we modeled cohort breeding data through development of a Monte Carlo simulator with an automated graph plotter (details of the program and instructions for use are available at: https://github.com/roshnigiyer/Monte_Carlo_Simulator). The model’s parameters, which are derived from *H*. *robusta* life history data, include mean and standard deviation measures for time-to-first-clutch, inter-clutch intervals, clutch size, number of clutches laid, days survived after last clutch and lifespan for both *H*. *robusta* reared in isolation and in cohorts. In one set of simulations, the program probabilistically generated data for mean, standard deviation, minimum, maximum and 95% confidence interval values corresponding to the model’s parameters using data from the pool of 16 *H*. *robusta* reared in isolation.

Our Monte Carlo simulator generates these data in under 1 second on average using 100 simulation runs. In a second set of simulations, the corresponding values were generated using parameters inferred from the cohort of 48 *H*. *robusta*. Our Monte Carlo simulator generates these data in under 1.5 seconds on average using 100 simulation runs. The simulations also allowed us to match the size of the simulated cohorts to those of experimental cohorts.

**Fig 7:**
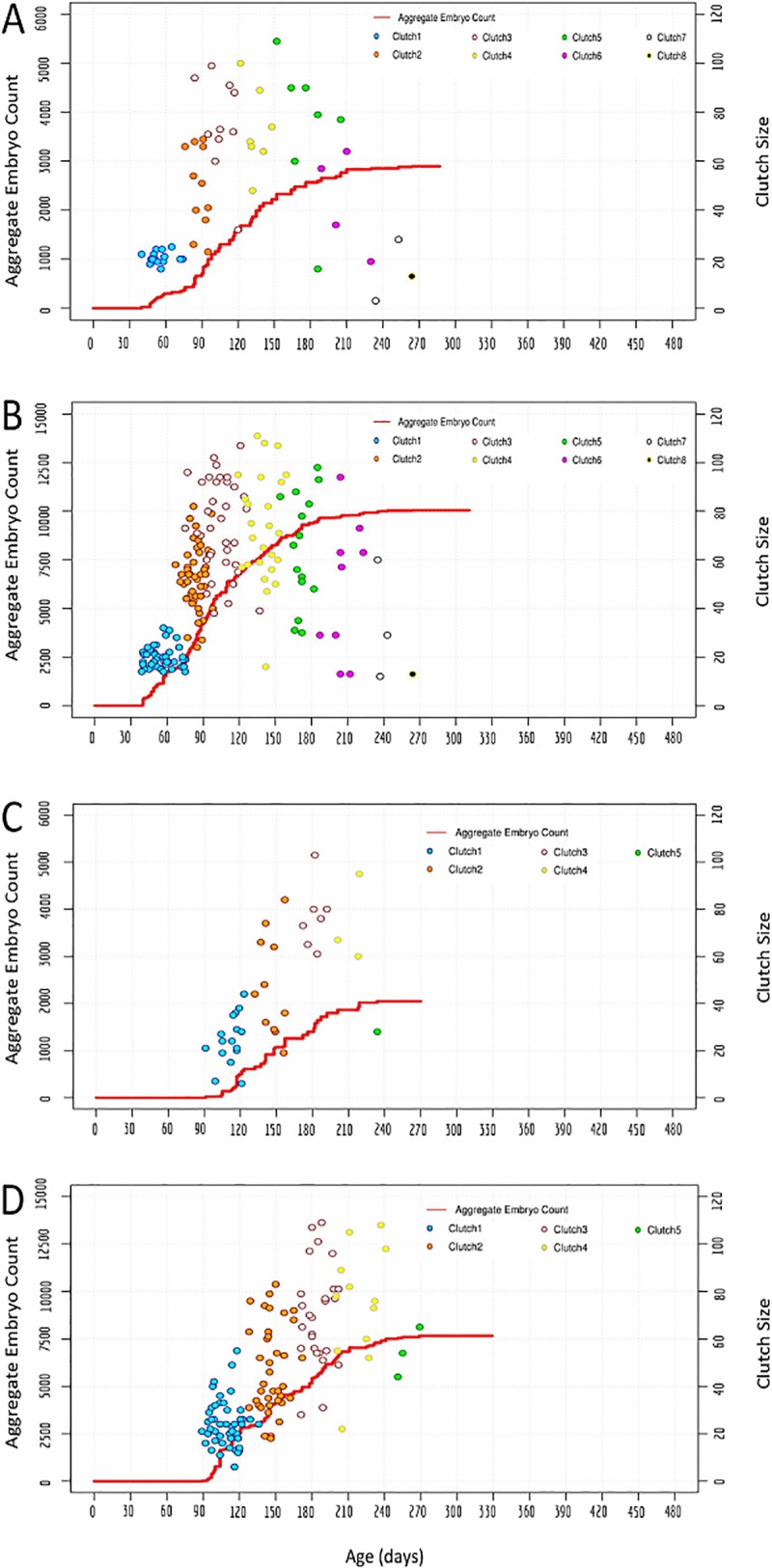
Monte Carlo simulations of reproductive activity in *H*. *robusta*. A, B) Simulated reproductivity of 16 and 48 animals, respectively, using parameters from 16 self-fertilizing animals raised in isolation. C, D) Simulation of 16 and 48 animals, respectively, using parameters inferred from the cohort of 48 interbreeding animals. Note that none of these simulations capture the temporal clustering observed in the experimental cohort of 48 animals.

The first set of simulations, based on parameters derived from individuals raised in isolation, accurately reproduced behavioral activity of the pseudo-cohort as expected (compare Figs 6D and 7A), but failed to fully reproduce that of the true cohort (compare Figs 4C and 7B). The first bout of reproductive activity was clustered (indicating a low variance in the zygote-to-first-clutch generation time), but occurred much earlier than we observed in the actual cohort experiment. Repeated simulations for cohorts of either 16 or 48 individuals failed to produce the tightly clustered bouts of reproduction that had been observed throughout the actual cohort experiment, indicating that the variance of the inter-clutch intervals was higher for the individuals than for the cohort. Also as expected, the overall productivity of the simulated cohort of 48 animals was higher than was observed for the actual cohort of 48 animals.

The second set of simulations used parameters inferred from the cohort of 48 *H*. *robusta*. As expected, these simulations performed better in reproducing the zygote-to-first clutch generation time for the cohort, but still failed to capture the temporally clustered bout of reproduction observed in the true cohort (compare Figs 7D and 4C), indicating that the variance of the inferred inter-clutch intervals was higher than that of the actual inter-clutch intervals. We interpret this discrepancy as revealing that one or more animals counted as present at the beginning of a bout of reproduction either died or otherwise failed to reproduce, so that in counting up the clutches and assigning them to what we defined as one round of reproduction actually included clutches that were part of the subsequent round. As expected, simulating cohorts of 16 animals using parameters inferred from the cohort of 48 animals predicted lower productivity and also a tighter temporal clustering of reproductive activity than was obtained with the pseudo-cohort of 16 animals (compare Figs 7C and 6D). Thus, we conclude that the parameters for reproductive behavior of isolated *H*. *robusta* cannot account for the coordinated reproductive behavior exhibited by cohorts of interbreeding individuals.

## Conclusions

The work presented here examines the reproductive life histories, under laboratory conditions, of three glossiphoniid leech species in the genus *Helobdella*: *H*. *austinensis*, *H*. *octatestisaca* and *H*. *robusta*. This work complements a previous description of reproductive behavior for a fourth species, *H*. *triserialis* (Wedeen et al. 1990 [18]). The behavior of organisms in the laboratory cannot be held equivalent to their behavior in the field. Nonetheless, the fact that the reproductive differences were observed for animals under closely similar conditions provides evidence for differences among the taxa we studied.

### Interspecies differences in reproductive life history

All sexually reproducing clitellate annelids (oligochaetes and leeches) are simultaneous hermaphrodites, but self-fertilization is rare (or at least has not been widely observed). Self-fertilization has been reported for the piscolid leech species *Zeylanicobdella arugamensis* [21] and for the glossiphoniid leeches *Clepsine marginata* [23], *Helobdella triserialis* [18], and *H*. *papillornata* [22]—this latter species is apparently identical to the previously described *H*. *europea* [41]. We have previously speculated [25] that a capacity for self-fertilization may contribute to the species richness of the genus *Helobdella* (see below)—if self-fertilization can rescue genomic rearrangements that would result in otherwise infertile individuals, it would result in reproductive isolation of nascent species without significant changes in habit or habitat (sympatric speciation).

One major distinction in reproductive life strategies for both plants and animals is whether individuals of a species reproduce only once (semelparity) or more than once (iteroparity) before dying. Semelparity has been documented for several glossiphoniid leech species including *Alboglossiphonia polypompholyx* [42], *Marsupiobdella africana* [43], *Theromyzon cooperei* [44], *T*. *rude* and *T*. *tessulatum* [45]. In contrast, iteroparity holds for several other leech species, e.g., the medicinal leech *Hirudo medicinalis* [46].

Based on the work presented here, iteroparity and the capacity for self-fertilization appear to be the rule in the genus *Helobdella*, but our work reveals a number of species-specific differences in reproductive capacity. Under similar laboratory conditions, three species we have studied, *H*. *octatestisaca*, and *H*. *robusta* in the present work, and *H*. *triserialis* in previous work are all self-fertile. Studied as isolated individuals, there were clear differences in reproductive behavior among these three species under our laboratory conditions: *H*. *octatestisaca* individuals never produced more than three clutches of cocoons, *H*. *triserialis* routinely produced five clutches, but never more, and *H*. *robusta* produced up to eight clutches (Tables 1 and 2; S2, S3 and S4 Tables).

In previous studies, reproductive life histories for *Helobdella* have been inferred from systematic measurements of size and reproductive status of wild-caught animals at different times of year for species identified as *H*. *stagnalis* in Wales [47,48] and in Tunisia [49]. The conclusion of these studies was that *H*. *stagnalis* undergoes two rounds of reproduction in the field.

*Helobdella* is a speciose genus compared with other groups of glossiphoniid leeches [15]. As has proven to be the case for some other widespread taxa, molecular sequence analyses have led to identification of cryptic species [14]. At one point, all leeches bearing a nuchal scute (Fig 1C), were classified as *H*. *stagnalis*, but there now appear to more than a dozen such species [15, 28, 29]. Based on its CO1 sequence, the *H*. *stagnalis*-like species we studied here is *H*. *octatestisaca*, originally described from Taiwan but apparently introduced there from Mexico [20]; its presence in California could represent a broader natural range than previously thought, perhaps thanks to migratory waterfowl or introduction by humans. The difference in the degree of iteroparity between *H*. *robusta* or *H*. *triserialis* as opposed to *H*. *stagnalis* or *H*. *octatestisaca* is consistent with tendency to divide the genus into distinct “*H*. *stagnalis*” and “*H*. *triserialis*” complexes, based on the presence or absence of the nuchal scute, respectively [15, 16, 29].

In fact, however, the *H*. *stagnalis*-like species are paraphyletic with respect to other *Helobdella* species on the basis of cytochrome oxidase 1 (CO1) sequence alone [15,16] (S1 Fig). Whether this reflect the limited number of informative sites in the CO1 sequence remains to be determined. Whether the difference between producing two clutches (for the morphologically defined *H*. *stagnalis* in Wales and Tunisia) or three clutches (for *H*. *stagnalis*-like species we identify as *H*. *octatestisaca*) reflect genuine inter-species differences, differences between laboratory and field conditions, or differences in the precision of field versus lab studies, also remains open. In any case, our results also show a significant difference in iteroparity among *H*. *austinensis*, *H*. *robusta* and *H*. *triserialis*, all belonging to the nominal *triserialis* complex, which indicates the lability of this trait within the genus.

### Inferring reproductive parameters from interbreeding cohorts

Based on our CO1 sequence analysis, the four species of *Helobdella* currently known to be self-fertile represent three distinct, well-supported clades: one containing *H*. *triserialis* [18] and *H*. *papillornata/europea* [22]; another containing *H*. *robusta*; and a third containing *H*. *octatestisaca* (S1 Fig; S1 Table). Two taxa within the outgroup for this tree, the glossiphoniid species *Hemiclepsis marginata* [23] and the piscicolid species *Zeylanicobdella arugamensis* [21] are also self-fertile. Thus, while the deeper branches of the *Helobdella* CO1 tree are not well-supported, it is parsimonious to suggest that the capacity for self-fertilization is ancestral within the genus *Helobdella* at least. Thus, we were surprised to find that *H*. *austinensis* appears incapable of reproducing by self-fertilization, especially since this species falls within the well-supported clade that includes the self-fertile *H*. *robusta*.

To infer the reproductive behavior of individuals in this species, we followed two interbreeding cohorts throughout their entire lifespans, and concluded that this species also produces a maximum of three clutches of embryos (Figs 2 and 3). These inferences were drawn based on the basic assumption that the reproductive behavior of animals within the cohort is approximately the same. We note that inter-animal variations in the distribution of surface markings such as papilla and pigment cells in *H*. *austinensis* should make it possible to identify and distinguish individual animals [24]. In principle, such morphological heterogeneity could make it possible to track the reproductive behavior of individuals within a cohort directly. Such an undertaking was beyond the scope of the present work, however.

Comparing the reproductive parameters of the two cohorts of *H*. *austinensis* revealed that the temporal features of reproductive activity were well conserved between the two experiments, as judged by both the distribution of egg-to-egg generation times and the inferred inter-clutch intervals (Table 2). In contrast, the reproductive capacity differed markedly between the two cohorts, averaging 156 zygotes per individual in the smaller cohort (starting with 23 individuals), compared with only 101 zygotes per individual in the larger cohort (starting with 60 individuals). This difference cannot be explained by differences in cohort survival—in both experiments, many animals survived for weeks after the cessation of reproductive activity, and the larger cohort deposited more cocoons overall; rather, the first and second clutches for the smaller cohort averaged more than twice the size of those in the larger cohort.

Both cohorts were raised in the same size of container. Thus, the population density was higher for the larger cohort than for the smaller one. In this context, difference in the progeny produced is consistent with theoretical predictions and experimental observations on the population density-dependence of sperm competition and reproductive resource allocation in simultaneous hermaphrodites [50–52]. In brief, and taking the extreme case of a single self-fertile hermaphrodite, the optimal reproductive strategy for such an individual would be to make as many eggs as energetically feasible, and restrict sperm production to the bare minimum required to fertilize those eggs. In contrast, as the population density increases, and thus the probability of interbreeding instead of self-fertilization, it is advantageous to make more sperm, in the expectation of being able to fertilize eggs from another individual, and fewer of the energetically more costly eggs, which are more likely to be fertilized by another individual.

These ideas have been tested in various species, including experiments with the leech species *H*. *papillornata*/*H*. *europea* [22]. Using total volume of testisacs and eggs as proxies for investment in sperm and eggs, respectively, these authors found that normalized testisac volume increased with increasing group size, but that egg volume did not. Our experiments do not permit statistical tests, but do suggest that for iteroparous species, measuring differences in overall egg production during the reproductive life of the individual, rather than at a single time point, might also reveal plasticity in maternal as well as paternal investment at different population densities.

Absent experimental replicates of the cohort size effects, which are beyond the scope of the present work, the preceding comments are clearly in the realm of speculation. We suggest, however, that *Helobdella* may be a valuable experimental system for testing theoretical prediction of population density effects and other aspects of reproductive resource allocation.

### Intra-species differences between reproduction by self-fertilization and interbreeding

To look for differences between the reproductive behavior of self-fertilizing and interbreeding individuals within the same species, we also followed the reproductive behavior of a *H*. *robusta* cohort. This experiment yielded three noteworthy results.

First, animals in the cohort exhibited a significant delay in the onset of reproduction compared to individuals reared in isolation (107.4 +/- 10.7 days vs. 56.3 +/- 8.7 days). This result differs from observations on *H*. *papillornata* by Tan et al. [22], who reported that “Self-fertilization is possible, because isolated individuals have produced offspring in the laboratory, but our observations suggest that individuals resort to self-fertilization only after a long period in which no partners could be found.” Notwithstanding these observations of delayed reproduction in isolated *H papillornata*, it would also seem reasonable for isolated self-fertile animals to initiate reproductive activity as soon as possible, to increase the population size—thereby increasing the probability of surviving the population bottleneck, and also enabling dispersal to increase the chances for encountering other conspecifics for subsequent interbreeding.

Second, the reproductive capacity of *H*. *robusta* in the cohort (averaging 149 zygotes per individual) was much lower than those raised in isolation (averaging 267 zygotes per individual). In contrast to our observations for *H*. *austinensis*, however, the difference between self-fertilizing and interbreeding *H*. *robusta* arises from differences in the numbers of clutches produced, and not from differences in clutch size. Self-fertilizing animals produced an average of 3.7 clutches per individual, with an observed maximum of eight, whereas the cohort-reared animals produced an average of 2.4 clutches each, with an inferred maximum of five. The difference in reproductive capacity is again consistent with the predictions of reproductive resource allocation theory. In this case however, as noted above, it is also possible that more animals in the cohort died before exhausting their reproductive capacity.

A final intriguing difference between the reproductive behavior of *H*. *robusta* in isolation, as opposed to an interbreeding cohort, is the clustering of reproductive episodes among individuals in the cohort. Monte Carlo simulations confirm that this clustering reflects a tightly distributed timing of reproductive episodes which cannot be explained based on the reproductive behavior of animals in isolation (Figs 4, 6 and 7). Precisely synchronized reproduction is well-known in certain marine polychaetes, providing the advantages of increased probability for encountering mates and overwhelming predators by mass producing spawn [53, 54], but has not been noted for leeches.

A possible model to explain this clustered reproductive behavior starts with the notion that maternity is much more costly than paternity for glossiphoniid leeches, whose reproduction involves a large maternal investment: first, cross-fertilization is by traumatic insemination, in which spermatophores implanted into the body wall of the partner digest their way through the multiple layers of the body wall before releasing sperm into the coelom [1]; in addition, glossiphoniid leeches make large, yolk-rich eggs, brood their embryos in cocoons attached to the ventral aspect of the parent and carry the juveniles with them to the first one or more feedings. Given the high cost of the maternal role for these hermaphrodites, it would seem advantageous for individuals in a cohort to retard maturation of their eggs until others in the cohort are susceptible to being sperm acceptors as well as sperm donors, thereby balancing out the physiological costs of maternity with the advantages of paternity.

We speculate that this model could account for both the delayed onset of reproductive activity in the cohort relative to the isolated individuals, and for the clustered reproductive activity exhibited by cohorts of *H*. *robusta* relative to individuals. It remains to be seen whether this clustered reproductive activity observed in laboratory conditions has ramifications for natural populations, which we imagine to be at lower densities and less well synchronized developmentally. But in any case, it seems clear that various *Helobdella* species provide a phenomenologically rich, experimentally tractable resource for investigating reproductive life history strategies and resource allocation by simultaneous hermaphrodites.

## Supporting information

S1 Fig Molecular phylogeny (Maximum Likelihood tree) of selected *Helobdella* and outgroup species based on partial CO1 sequence; boxes enclose the three taxa studied here. Taxa known to be capable of reproduction by self-fertilization are in blue; taxon known to be incapable of reproduction by self-fertilization is in red. Breaks indicate long branches that were halved to conserve space. Green asterisks indicate scute-bearing (stagnalis-like) taxa. Branch support scores are from SH-like approximate likelihood ratio tests; only values ≥ 50% are shown. Branch lengths are proportional to molecular change (amino acid substitutions/site) between nodes; see scale bar for measurement. References and accession numbers are provided in S1 Table.
(PDF)

S1 Table Details of *Helobdella* taxa [55–59] and close outgroups [60–62] included in S1 Fig. (PDF)

S2 Table Summary of reproductive life history data for individual *Helobdella triserialis*, raised in isolation on snail diet (from [18]). Reproductive life histories were obtained for individual leeches raised in isolation and fed on snails, similar to the procedures used in the present study. No individual laid more than five clutches of embryos, despite living for as long as 100 days after the last laying. The egg-to-egg generation time is denoted by the interval between deposition of the zygote from which a given animal developed and the deposition of the first clutch of embryos by that animal (ZD-C1). Subsequent inter-clutch intervals are denoted as C1-C2, C2-C3, C3-C4 and C4-C5, respectively. For each category, the sample size is indicated by (N).
(PDF)

S3 Table Detailed reproductive life history data for 16 individual, self-fertilizing *Helobdella robusta*, raised in isolation on snail diet. Data presentation and abbreviations are as in S2 Table. Columns A through H denote inter-clutch intervals as follows: A, ZD-C1 (15); B, C1-C2 (15); C, C2-C3 (14); D, C3-C4 (12); E, C4-C5 (10); F, C5-C6 (8); G, C6-C7 (5); H, C7-C8 (2). Columns I through P denote clutch sizes as follows: I, C1 (10); J, C2 (14); K, C3 (14); L, C4 (11); M, C5 (11); N, C6 (9); O, C7 (6); P, C8 (1).
(PDF)

S4 Table Detailed reproductive life history data for 5 individual, self-fertilizing *Helobdella octatestisaca*, raised in isolation on bloodworm diet. Abbreviations and data organization as in S3 Table.
(PDF)
