# Supplementary figures and images for: Reproductive differences among species, and between individuals and cohorts, in the leech genus Helobdella (Lophotrochozoa; Annelida; Clitellata; Hirudinida; Glossiphoniidae), with implications for reproductive resource allocation in hermaphrodites
Source: PLoS One. 2019 Apr 1;14(4):e0214581. doi: 10.1371/journal.pone.0214581 (PMC6443171; doi:10.1371/journal.pone.0214581)

S1 Fig

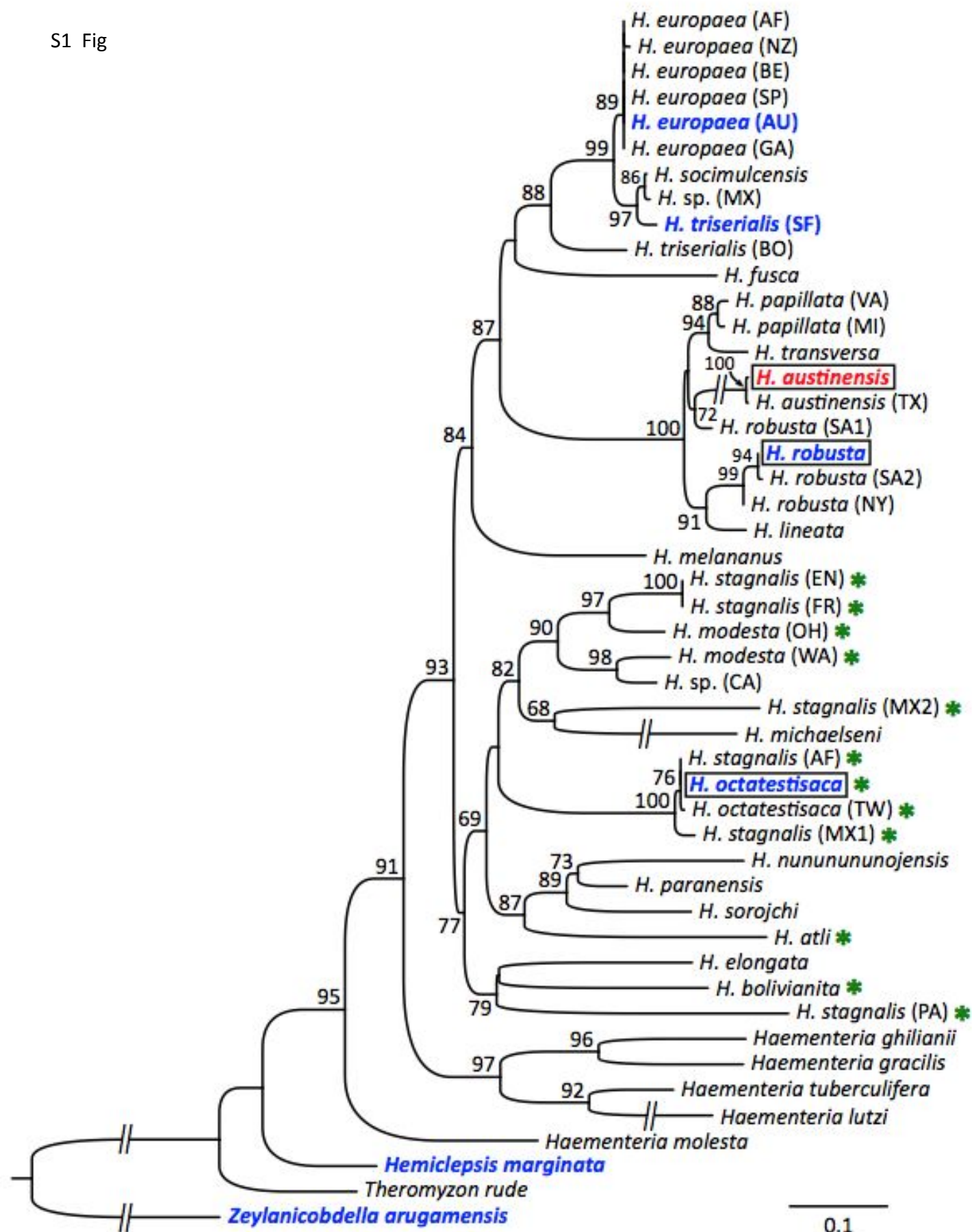

Supplement: S1 Fig — Taxa known to be capable of reproduction by self-fertilization are in blue; taxon known to be incapable of reproduction by self-fertilization is in red. Breaks indicate long branches that were halved to conserve space. Green asterisks indicate scute-bearing (stagnalis-like) taxa. Branch support scores are from SH-like approximate likelihood ratio tests; only values ≥ 50% are shown. Branch lengths are proportional to molecular change (amino acid substitutions/site) between nodes; see scale bar for measurement. References and accession numbers are provided in S1 Table. (PDF) [file pone.0214581.s001.pdf]
