# Supplementary material for: Reproductive differences among species, and between individuals and cohorts, in the leech genus Helobdella (Lophotrochozoa; Annelida; Clitellata; Hirudinida; Glossiphoniidae), with implications for reproductive resource allocation in hermaphrodites
Source: PLoS One. 2019 Apr 1;14(4):e0214581. doi: 10.1371/journal.pone.0214581 (PMC6443171; doi:10.1371/journal.pone.0214581)
Supplement: S1 Table — (PDF) [file pone.0214581.s002.pdf]

S1 Table

Species	Collection Site	GenBank Accession Number (CO1)	Reference
Helobdella			
H. atli	borough of Xochimilco, Mexico City, Mexico	HQ179852	Oceguera-Figueroa et al. 2010
H. austinensis	lab strain; originally from Austin, TX, USA	MH729328	This paper
H. austinensis (TX)	Austin, TX, USA	DQ995309	Bely & Weisblat, 2006
H. bolivianita	Bolivia	AF329053	Siddall & Borda, 2003
H. elongata	Michigan, USA	AF329045	Siddall & Borda, 2003
H. europaea (AF)	South Africa	AY856048	Siddall & Budinoff 2005
H. europaea (AU)	Magile Creek, Brisbane, Australia	AF329052	Siddall & Borda, 2003
H. europaea (BE)	Berkeley, CA, USA	DQ995304	Bely & Weisblat, 2006
H. europaea (GA)	Galt, CA, USA	DQ995297	Bely & Weisblat, 2006
H. europaea (NZ)	New Zealand	AY856049	Oceguera-Figueroa et al. 2010
H. europaea (SP)	Spain	KC904242	Reyes-Prieto et al. 2014
H. fusca	Wild Goose Lake, MI, USA	AF329038	Siddall & Borda, 2003
H. lineata	Douglas Lake, MI, USA	AF329039	Siddall & Borda, 2003
H. melananus	Taiwan	FJ000355	Lai et al. 2009
H. michaelseni	Chile	AF536824	Siddall & Borda, 2003
H. modesta (OH)	Columbus, OH, USA	AF329040	Siddall & Borda, 2003
H. modesta (WA)	Washington, USA	HQ179854	Oceguera-Figueroa et al. 2010
H. nununununojensis	Madidi, Bolivia	AF329048	Siddall & Borda, 2003
H. octatestisaca	lab strain; originally from Sacramento, CA, USA	MH729329	This paper
H. octatestisaca (TW)	Taiwan	FJ000348	Lai et al. 2009
H. papillata (MI)	Round Lake, MI, USA	AF329043	Siddall & Borda, 2003
H. papillata (VA)	Gloucester, VA, USA	AF329046	Siddall & Borda, 2003
H. paranensis	Arroyo Aspinas, Uruguay	AF329037	Siddall & Borda, 2003
H. robusta	lab strain; originally from Sacramento, CA, USA	MH729330	This paper
H. robusta (NY)	Tarrytown, NY, USA	DQ995305	Bely & Weisblat, 2006
H. robusta (SA1)	Sacramento, CA, USA	DQ995300	Bely & Weisblat, 2006
H. robusta (SA2)	Sacramento, CA, USA	AF178680	Boore & Brown, 2000
H. socimulcensis	Cuautla, Morelos, Mexico	HQ179872	Oceguera-Figueroa et al. 2010
H. sorojchi	Madidi, Bolivia	AF329050	Siddall & Borda, 2003
H. species (CA)	Sequoia National Park, California, USA	KF000158	Deiner et al. 2013

H. species (MX)	Mexico	DQ995311	Bely & Weisblat, 2006
H. stagnalis (AF)	South Africa	HQ179860	Oceguera-Figueroa et al. 2010
H. stagnalis (EN)	Costwolds, England	AF329041	Oceguera-Figueroa et al. 2010
H. stagnalis (FR)	France	AF116018	Apakupakul et al. 1999
H. stagnalis (MX1)	Ameca, Mexico	HQ179856	Oceguera-Figueroa et al. 2010
H. stagnalis (MX2)	Morelos, Mexico	HQ179861	Oceguera-Figueroa et al. 2010
H. stagnalis (PA)	Pennsylvania, USA	JN692269	Unpublished
H. transversa	Cheboygan State Park, Michigan, USA	AF329044	Siddall & Borda, 2003
H. triserialis (BO)	Laguna Volcan, Bolivia	AF329054	Siddall & Borda, 2003
H. triserialis (SF)	San Francisco, CA, USA	DQ995303	Bely & Weisblat, 2006
Outgroups			
Haementeria ghilianii	French Guiana	AF329035	Siddall & Borda, 2003
Haementeria gracilis	Arroyo Aspinas, Uruguay	AF329034	Siddall & Borda, 2003
Haementeria lutzi	Rio Pastaza, Ecuador	AF329033	Siddall & Borda, 2003
Haementeria molesta	Arroyo Aspinas, Uruguay	AF329469	Siddall & Borda, 2003
Haementeria tuberculifera	Iquitos, Peru	JN850910	Oceguera-Figueroa 2012
Hemiclepsis marginata	E´tang de La Musse, Paimpont, France	AF003259	Siddall & Burreson, 1998
Theromyzon rude	Lake of Two Rivers, Algonquin Park, Ontario, Canada	AF003262	Siddall & Burreson, 1998
Zeylanicobdella arugamensis	Jerejak Island, Penang, Malaysia	KY441717	Ravi & Yahaya, 2017
