# Supplementary material for: Reproductive differences among species, and between individuals and cohorts, in the leech genus Helobdella (Lophotrochozoa; Annelida; Clitellata; Hirudinida; Glossiphoniidae), with implications for reproductive resource allocation in hermaphrodites
Source: PLoS One. 2019 Apr 1;14(4):e0214581. doi: 10.1371/journal.pone.0214581 (PMC6443171; doi:10.1371/journal.pone.0214581)
Supplement: S2 Table — Reproductive life histories were obtained for individual leeches raised in isolation and fed on snails, similar to the procedures used in the present study. No individual laid more than five clutches of embryos, despite living for as long as 100 days after the last laying. The egg-to-egg generation time is denoted by the interval between deposition of the zygote from which a given animal developed and the deposition of the first clutch of embryos by that animal (ZD-C1). Subsequent inter-clutch intervals are denoted as C1-C2, C2-C3, C3-C4 and C4-C5, respectively. For each category, the sample size is indicated by (N). (PDF) [file pone.0214581.s003.pdf]

S2 Table. ***H.triserialis*** **self-fertilizing, snail diet (from Weeden et al 1980)**

	ZD-C1 (16)	C1-C2 (15)	C2-C3 (12)	C3-C4 (8)	C4-C5 (4)	C1 (15)	C2 (13)	C3 (12)	C4 (9)	C5 (6)
**Average inter-clutch interval in days**	69 +/- 9	99 +/- 14	131 +/- 18	165 +/- 23	200 +/- 26					
**Average clutch size**						43 +/- 16	99 +/- 36	133 +/- 58	101 +/- 54	67 +/- 26
**Clutch size range**						[17, 65]	[49, 166]	[44, 228]	[15, 198]	[24, 101]
