# Supplementary material for: Reproductive differences among species, and between individuals and cohorts, in the leech genus Helobdella (Lophotrochozoa; Annelida; Clitellata; Hirudinida; Glossiphoniidae), with implications for reproductive resource allocation in hermaphrodites
Source: PLoS One. 2019 Apr 1;14(4):e0214581. doi: 10.1371/journal.pone.0214581 (PMC6443171; doi:10.1371/journal.pone.0214581)
Supplement: S4 Table — Abbreviations and data organization as in S3 Table. (PDF) [file pone.0214581.s005.pdf]

S4 Table. ***H. octitestasaca*****, self-fertilizing, bloodworm diet (N = 5)**

		ZD-C1 (5)	C1-C2 (4)	C2-C3 (3)	C1 (5)	C2 (5)	C3 (3)
**Lifespan (5)***	246 +/- 60						
**Lifespan range (5)***	[181, 325]						
**Post-repro. life (5)***	59 +/- 47						
**Post-repro. life range (5)***	[16, 125]						
**Generation time (5)***	140 +/- 26						
**Generation time range (5)***	[120, 180]						
**Inter-clutch interval**		140 +/- 26	161 +/- 38	221 +/- 43			
**Inter-clutch interval range**		[120, 180]	[137, 227]	[191, 270]			
**Clutch size**					26 +/- 8	50 +/- 13	70 +/- 5
**Clutch size range**					[17, 37]	[33, 66]	[65, 74]
**Total embryo production (5)***	119 +/- 36						
**Embryo production range (5)***	[67, 160]						
